# Research on the equilibrium strategy of data value co-creation in water conservancy engineering projects based on differential game theory

**DOI:** 10.1371/journal.pone.0342024

**Published:** 2026-06-29

**Authors:** Xiaowei An, Xi Chen, Guanghua Dong, Lingli Zhang

**Affiliations:** North China University of Water Resources and Electric Power, Zhengzhou, Henan, China; The University of Jordan Faculty of Business: The University of Jordan School of Business, JORDAN

## Abstract

Water conservancy engineering projects are characterized by long construction cycles, multiple participants, and dispersed data, resulting in low efficiency in data resource utilization. Therefore, data value co-creation is needed to enhance the value of data elements. This study develops a three-party dynamic differential game model of data value co-creation involving the contractor, designer, and owner. Both centralized and decentralized decision-making scenarios are considered, and a government subsidy mechanism is introduced to analyze multi-party collaborative decision-making behavior and its influencing factors. The results show that: (1) Under the centralized decision-making scenario, both the data value level and the total returns from data value co-creation are higher than those under the decentralized decision-making scenario. Centralized decision-making is more conducive to maximizing data value. (2) Government subsidies can increase the level of effort and returns of participating subjects, thereby incentivizing these subjects to engage in data value co-creation. (3) The cost coefficient of data value co-creation for participating parties has an inhibitory effect on their willingness to engage in data value co-creation. In contrast, an increase in the utility coefficient can significantly enhance their willingness. The research findings can provide theoretical support for data value co-creation practices in water conservancy projects and for the formulation of related policies.

## 1. Introduction

Water conservancy projects are important infrastructure for economic and social development, and the construction quality is directly related to water resource security, coordinated regional economic development, and national ecological security [1]. With the development of technology and industrial transformation, digital and intelligent technologies such as big data, the Internet of Things (IoT), and artificial intelligence (AI) are increasingly integrated into water conservancy projects [2]. This integration is driving the transformation of water conservancy project construction from traditional experience-driven approaches to digital-driven approaches [3,4]. Water conservancy projects are characterized by large construction scales and long construction periods. The construction process generates massive amounts of data, which have potential value for optimizing resource allocation, reducing construction costs, and enhancing engineering resilience [5,6]. The effective utilization of data and the full release of data value can help realize the refined management of the entire life cycle of water conservancy projects, and promote the digital transformation of water conservancy projects [7,8]. This plays an irreplaceable role in improving the comprehensive benefits of water conservancy projects.

However, water conservancy projects involve numerous stakeholders, and the data generated during project construction is often dispersed among different parties, such as designers, contractors, and owners. The fragmented state of data constrains the effective utilization of data resources and the realization of data value [9]. Therefore, data value co-creation has gradually emerged as an important approach to promoting the transformation of water conservancy project data resources into tangible value outcomes [10,11]. Data value co-creation emphasizes collaborative interaction among multiple stakeholders around data resources. Such collaboration enables value enhancement through continuous data integration and resource consolidation [12,13]. However, in the practice of data value co-creation in water conservancy projects, the willingness of participating parties is generally weak and the depth of collaboration is insufficient. Consequently, the outcomes of data value co-creation are often unsatisfactory [14]. Different subjects have different interest demands, and there are certain differences in their cognition of data value and utilization goals [15,16]. This will not only exacerbate the data silo phenomenon but also may trigger free-riding behavior [17]. Therefore, to clarify the causes of this issue, it is necessary to analyze the evolutionary process of multi-party data value co-creation behavior from the perspective of dynamic strategy selection [18]. This study develops a dynamic evolutionary model for data value co-creation in water conservancy projects based on differential game theory. It examines the equilibrium strategies of multi-party data value co-creation and their influencing mechanisms, with the aim of providing support for data value co-creation practices in water conservancy projects.

The remainder of this study is organized as follows. Section 2 presents the literature review. Section 3 develops the differential game model for data value co-creation. Section 4 provides the solution process of the model. Section 5 analyzes the evolution of equilibrium strategies for data value co-creation in water conservancy project construction and examines the effects of key factors on the game results through numerical simulations. Section 6 discusses the findings and presents policy implications. The final section presents the conclusions.

## 2. Literature review

With the rapid development of the digital economy, data has gradually become an important factor of production, and its value has attracted widespread attention [19]. Existing studies have analyzed the mechanisms underlying data value formation from the perspective of data resources. Bonvino and Giorgino [20] propose a data valorization framework from a strategic management perspective, emphasizing value creation through data orchestration and collaborative management. Feng and Liu [21] point out that digital transformation and the upgrading of innovation capabilities can promote the utilization of data resources and enhance value creation capacity. Zhang et al. [22] investigate collaborative innovation mechanisms under a data-driven model. Their findings indicate that data integration can effectively improve collaborative innovation performance among multiple actors. Benmohamed et al. [23] show that open government data can promote public value creation. However, its realization depends on organizational support and data utilization capabilities within government institutions. These studies suggest that the integration and utilization of data resources enhance value creation capacity, thereby providing a theoretical foundation for research on data value. Building on this foundation, scholars have further advanced the concept of data value co-creation [24]. Relevant studies indicate that collaborative interactions and data sharing among multiple actors are important conditions for realizing data value co-creation. Volz et al. [25] emphasize the supporting role of multi-party data resource integration capability in data value creation within digital ecosystems. Kamalaldin et al. [26] argue that changes in the structure of relationships among actors influence the value co-creation process. Liu et al. [27] analyze that interaction intensity among actors significantly influences the level of value creation. Fang et al. [28] examine the issue from the perspective of an open government data ecosystem. They suggest that the collaborative participation of multiple actors, such as governments, enterprises, and the public, is a key condition for realizing data value. Meanwhile, Wang et al. [29] point out that the integration of multi-source data can promote information sharing and value creation. Furthermore, Lin et al. [30] reveal the value co-creation strategies of multiple stakeholders and their influencing factors within the digital servitization ecosystem based on an evolutionary game model. Overall, existing studies have explored the mechanisms of data value co-creation from the perspectives of multi-actor interaction and strategic evolution. However, most of this research focuses on fields such as digital services, manufacturing, and open government data. Relatively little attention has been paid to data value co-creation in the construction sector.

In the construction sector, with the widespread application of technologies such as BIM, digital twins, and artificial intelligence, engineering data has gradually become an important resource for improving project performance and management efficiency [31,32]. Han and Li [33] point out that the large volume of data generated throughout the lifecycle of engineering projects can support project decision-making and management optimization, thereby improving project management efficiency. Wang et al. [34] investigate barriers and promotion strategies for data sharing in civil infrastructure projects. Their study highlights that data sharing is an important approach to improving the level of project collaboration and value creation. Hua et al. [35] analyze that digital capabilities significantly enhance innovation performance in engineering projects and promote collaborative value creation. Keskin et al. [36] examine collaboration mechanisms in airport project delivery from the perspective of BIM technology ecosystems. They find that digital technologies facilitate multi-stakeholder collaboration in engineering projects. In addition, some scholars have examined data value co-creation in engineering projects from the perspective of strategic interaction. Cidik and Bowler [16] investigate the value formation process in construction projects from a project practice perspective. Their findings indicate that value co-creation emerges through interactive practices among multiple stakeholders. Liang and Song [37] analyze collaborative paths in EPC projects from the perspective of value co-creation behavior networks. They show that multi-stakeholder collaboration is a key driving force for value co-creation in engineering projects. Wang et al. [10] study multi-stakeholder value co-creation strategies in the servitization transformation of the construction industry based on an evolutionary game model. Their results reveal the role of cooperation mechanisms in maintaining the stability of value co-creation. An et al. [38] also use an evolutionary game model to analyze data value co-creation behavior in engineering projects. Their study explores the evolution of strategies for multiple stakeholders participating in value co-creation. Overall, existing studies have examined value co-creation in the construction sector from the perspectives of project collaboration mechanisms and strategy evolution. However, most studies focus on collaboration mechanisms or behavioral evolution. Systematic research on data value co-creation in engineering project contexts remains limited. In particular, there is a lack of studies on multi-stakeholder effort investment, benefit distribution, and their dynamic equilibrium strategies.

**Fig 1:**
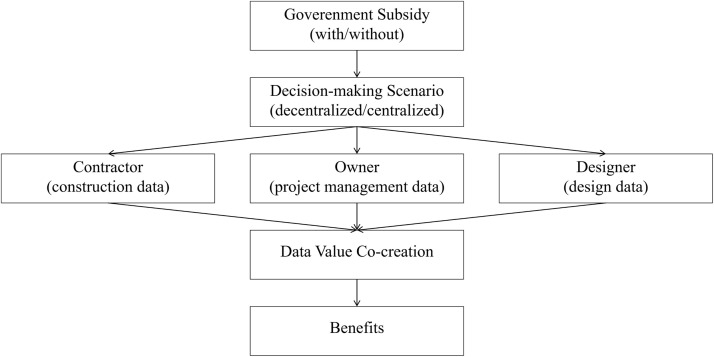
Analytical framework for data value co-creation in water conservancy projects.

A review of the existing literature shows that studies on data value co-creation primarily focus on data resource integration, multi-party collaboration, and strategy evolution, with preliminary applications in the engineering construction domain. However, few studies have investigated the dynamic decision-making mechanisms and equilibrium strategies of multi-party data value co-creation in the context of water conservancy projects. Therefore, this study considers the characteristics of water conservancy projects and incorporates government subsidies and different decision-making scenarios. A three-party differential game model involving contractor, designer, and owner is constructed to analyze the dynamic decision-making process of multi-party data value co-creation. The analytical framework of data value co-creation in water conservancy projects is shown in Fig 1.

The main innovations and contributions of this study are as follows. (1) From the perspective of water conservancy projects, this study investigates data value co-creation and data value mining involving multiple participating parties. This enriches research on data value co-creation in the water conservancy domain. (2) This study constructs a tripartite differential game model involving contractor, designer, and owner. By analyzing the equilibrium strategies of multi-party data value co-creation under different decision-making scenarios, it reveals the dynamic evolutionary patterns and influencing mechanisms of multi-party data value co-creation behavior. (3) This study examines the effects of key factors on data value co-creation strategies in water conservancy projects. The results provide theoretical support for data value co-creation practices and related policy formulation in water conservancy projects.

## 3. Construction of a differential game for data value co-creation

Water conservancy project construction involves multiple participants, and a large amount of data is dispersed among the contractor, designer, and owner. The level of data sharing and collaborative behaviors directly affects the effectiveness of data value co-creation [39]. Therefore, this study selects the contractor, designer, and owner as the three parties to analyze the equilibrium strategies of data value co-creation in water conservancy projects. Due to the long construction cycle of water conservancy projects, project data are continuously generated during the construction period and exhibit dynamic evolutionary characteristics. Therefore, this study employs a differential game model to characterize the dynamic decision-making process of data value co-creation among the parties [40].

During the construction of water conservancy projects, the contractor, designer, and owner are all characterized by bounded rationality. Aiming to facilitate data value co-creation in water conservancy projects, this study develops a tripartite differential game model involving the contractor, designer and owner. Through solving for the equilibrium solutions of the game model, the equilibrium strategies for data value co-creation are analyzed. Meanwhile, the key influencing factors in the construction process of data value co-creation are identified, and the influence of key factors on the game results is explored.

### 3.1. Model assumptions

*Assumption 1* In the process of data value co-creation in water conservancy project construction, the effort levels of contractor, designer and owner are denoted as X(t), Y(t), Z(t), which are specifically reflected in the levels of effort devoted to data collection, integration, and sharing. It is assumed that the costs of data value co-creation for contractor, designer and owner are $E_{C}$, $E_{D}$ and $E_{O}$. Generally, the cost incurred by participating parties in the value co-creation process is positively correlated with their effort levels; thus, it can be assumed that $E_{C}=\frac{\text{1}}{\text{2}}\alpha_{C}X^{\text{2}}\left(t\right)$, $E_{D}=\frac{\text{1}}{\text{2}}\alpha_{D}Y^{\text{2}}\left(t\right)$, $E_{O}=\frac{\text{1}}{\text{2}}\alpha_{O}Z^{\text{2}}\left(t\right)$ [41], where $\alpha_{C}$, $\alpha_{D}$ and $\alpha_{O}$ represent the cost coefficients of data value co-creation for contractor, designer and owner. During the construction of engineering projects, these costs mainly arise from investments in multi-stage data collection, processing, sharing, and data integration applications.

*Assumption 2* Contractor, designer and owner conduct collaborative data integration, thereby enhancing data value. Let the data value level be denoted as I(t), which is determined by the data value co-creation effort levels of the contractor, designer and owner. The data value level changes over time and can be described by the following differential equation [42]:


$$\begin{array}{c} \mathrm{\backslash stackrel}⬝I\left(t\right)=\beta_{C}X\left(t\right)\text{+}\beta_{D}Y\left(t\right)+\beta_{O}Z\left(t\right)-\delta I\left(t\right) \end{array}$$ (1)

In Eq.(1), $\beta_{C}$, $\beta_{D}$ and $\beta_{O}$ denotes the utility coefficient of the data value level for contractor, designer and owner at time t. Considering the long construction cycle of water conservancy projects and the high uncertainty in the construction process, the timeliness of data value is significant. Therefore, this study introduces a data value decay coefficient δ to characterize the dynamic evolution of data value in water conservancy projects, and δ>0 [43]. Let the initial data value level be denoted as $I_{\text{0}}$, and $I_{\text{0}}\geq \text{0}$.

*Assumption 3:* Water conservancy projects exhibit public attributes and multi-objective benefit characteristics. Data value co-creation can not only reduce project costs, shorten construction duration, and improve project quality, but also generate social and ecological benefits. Let the total returns of data value co-creation be denoted as R(t). The benefits of data value co-creation are positively related to data value and are commonly assumed to follow a linear relationship [40,41]. Therefore, R(t) can be set as:


$$\begin{array}{c} R\left(t\right)=\theta I\left(t\right) \end{array}$$ (2)

In Eq.(2), θ denotes the utility coefficient for converting data value into returns.

*Assumption 4:* The total returns obtained from data value co-creation by the contractor, designer, and owner are distributed among the three parties. The allocation ratios are denoted as $\xi_{\text{1}}$, $\xi_{\text{2}}$, $\xi_{\text{3}}$, $\xi_{\text{1}}\in \left[0,1\right]$, $\xi_{\text{2}}\in \left[0,1\right]$, $\xi_{\text{3}}\in \left[0,1\right]$, and $\xi_{\text{1}}\text{+}\xi_{\text{2}}\text{+}\xi_{\text{3}}\text{=1}$. At any time, contractor, designer and owner share the same discount rate ρ, and ρ>0 [44].

*Assumption 5:* Water conservancy project construction involves multiple participants, and their demands differ. As a result, decision-making objectives and logics vary among parties. Meanwhile, data are dispersed among different participants, leading to pronounced information asymmetry. Therefore, this study considers two decision-making scenarios: decentralized decision-making and centralized decision-making. Under decentralized decision-making, each participant makes decisions independently with the objective of maximizing its own benefit. Under centralized decision-making, the participants coordinate through a unified mechanism to maximize overall project benefits. In addition, water conservancy projects exhibit strong government-led characteristics. Accordingly, this study introduces a government subsidy mechanism to analyze the impact of policy incentives on data value co-creation [45,46]. Considering that centralized decision-making involves a higher degree of collaboration among participants, governments typically provide stronger policy incentives to promote data value co-creation. Therefore, it is assumed that the subsidy coefficient under centralized decision-making is higher than that under decentralized decision-making.

### 3.2. Decentralized decision-making scenario

In the decentralized decision-making scenario, the contractor, designer and owner, as independent decision-making subjects, make decisions independently to maximize their own interests.

Based on the aforementioned assumptions, the objective function of the contractor, denoted as $\overset{N}{\underset{C}{J}}$, is:


$$\begin{array}{c} \overset{N}{\underset{C}{J}}=\overset{\infty }{\underset{\text{0}}{\int }}e^{-\rho t}\left[\xi_{\text{1}}\theta I\left(t\right)-\frac{\text{1}}{\text{2}}\alpha_{C}X^{\text{2}}\left(t\right)\right]\text{d}t \end{array}$$ (3)

The objective function of the designer, denoted as $\overset{N}{\underset{D}{J}}$, is:


$$\begin{array}{c} \overset{N}{\underset{D}{J}}=\overset{\infty }{\underset{\text{0}}{\int }}e^{-\rho t}\left[\xi_{\text{2}}\theta I\left(t\right)-\frac{\text{1}}{\text{2}}\alpha_{D}Y^{\text{2}}\left(t\right)\right]\text{d}t \end{array}$$ (4)

The objective function of the project owner, denoted as $\overset{N}{\underset{O}{J}}$, is:


$$\begin{array}{c} \overset{N}{\underset{O}{J}}=\overset{\infty }{\underset{\text{0}}{\int }}e^{-\rho t}\left[\xi_{\text{3}}\theta I\left(t\right)-\frac{\text{1}}{\text{2}}\alpha_{O}Z^{\text{2}}\left(t\right)\right]\text{d}t \end{array}$$ (5)

### 3.3. Decentralized decision-making scenario with government subsidies

In the decentralized decision-making scenario with government subsidies, the project contractor, designer and owner are all independent decision-makers, each aiming to maximize their own interests. The government incentivizes all parties to participate in data value co-creation through subsidy policies. It is assumed that $\sigma_{\text{1}}$, $\sigma_{\text{2}}$ and $\sigma_{\text{3}}$ are the cost subsidy coefficients obtained by the contractor, designer and owner, respectively.

At this point, the objective function of the contractor, denoted as $\overset{S}{\underset{C}{J}}$, is:


$$\begin{array}{c} \overset{S}{\underset{C}{J}}=\overset{\infty }{\underset{\text{0}}{\int }}e^{-\rho t}\left[\xi_{\text{1}}\theta I\left(t\right)-\frac{\text{1}}{\text{2}}\alpha_{C}X^{\text{2}}\left(t\right)\text{+}\frac{\text{1}}{\text{2}}\sigma_{\text{1}}\alpha_{C}X^{\text{2}}\left(t\right)\right]\text{d}t \end{array}$$ (6)

The objective function of the designer, denoted as $\overset{S}{\underset{D}{J}}$, is:


$$\begin{array}{c} \overset{S}{\underset{D}{J}}=\overset{\infty }{\underset{\text{0}}{\int }}e^{-\rho t}\left[\xi_{\text{2}}\theta I\left(t\right)-\frac{\text{1}}{\text{2}}\alpha_{D}Y^{\text{2}}\left(t\right)\text{+}\frac{\text{1}}{\text{2}}\sigma_{\text{2}}\alpha_{D}Y^{\text{2}}\left(t\right)\right]\text{d}t \end{array}$$ (7)

The objective function of the owner, denoted as $\overset{S}{\underset{O}{J}}$, is:


$$\begin{array}{c} \overset{S}{\underset{O}{J}}=\overset{\infty }{\underset{\text{0}}{\int }}e^{-\rho t}\left[\xi_{\text{3}}\theta I\left(t\right)-\frac{\text{1}}{\text{2}}\alpha_{O}Z^{\text{2}}\left(t\right)\text{+}\frac{\text{1}}{\text{2}}\sigma_{\text{3}}\alpha_{O}Z^{\text{2}}\left(t\right)\right]\text{d}t \end{array}$$ (8)

### 3.4. Centralized decision-making scenario

Under the centralized decision-making scenario, the contractor, designer and owner achieve resource allocation and information sharing through a unified coordination mechanism. The three parties make decisions intending to maximize the overall benefit of data value co-creation.

Under the centralized decision-making scenario, the objective function, denoted as $J^{A}$, is:


$$\begin{array}{c} J^{A}=\overset{\infty }{\underset{\text{0}}{\int }}e^{-\rho t}[\theta I\left(t\right)-\frac{\text{1}}{\text{2}}\alpha_{C}X^{\text{2}}\left(t\right)-\frac{\text{1}}{\text{2}}\alpha_{D}Y^{\text{2}}\left(t\right)-\frac{\text{1}}{\text{2}}\alpha_{O}Z^{\text{2}}\left(t\right)]dt \end{array}$$ (9)

### 3.5. Centralized decision-making scenario with government subsidies

The government actively guides the contractor, designer and owner to collaborate in the process of data value co-creation through subsidy policies, so as to maximize the overall benefit. Let $\varepsilon_{\text{1}}$, $\varepsilon_{\text{2}}$ and $\varepsilon_{\text{3}}$ be the cost subsidy coefficients provided by the government to the contractor, designer and owner, respectively.

Under the centralized decision-making scenario with government subsidies, the objective function, denoted as $J^{T}$, is:


$$\begin{array}{c} J^{T}=\overset{\infty }{\underset{\text{0}}{\int }}e^{-\rho t}[\theta I\left(t\right)-\frac{\text{1}}{\text{2}}\alpha_{C}X^{\text{2}}\left(t\right)-\frac{\text{1}}{\text{2}}\alpha_{D}Y^{\text{2}}\left(t\right)-\frac{\text{1}}{\text{2}}\alpha_{O}Z^{\text{2}}\left(t\right)\text{+}\frac{\text{1}}{\text{2}}\varepsilon_{\text{1}}\alpha_{C}X^{\text{2}}\left(t\right)\text{+}\frac{\text{1}}{\text{2}}\varepsilon_{\text{2}}\alpha_{D}Y^{\text{2}}\left(t\right)\text{+}\frac{\text{1}}{\text{2}}\varepsilon_{\text{3}}\alpha_{O}Z^{\text{2}}\left(t\right)]\text{d}t \end{array}$$ (10)

The symbols and meanings of the parameters are shown in Table 1.

**Table 1:** Parameter definitions.

Parameter	Definition of parameters
X(t)	Contractor’s effort level
Y(t)	Designer’s effort level
Z(t)	Owner’s effort level
$\alpha_{C}$	Contractor’s cost coefficient for data value co-creation
$\alpha_{D}$	Designer’s cost coefficient for data value co-creation
$\alpha_{O}$	Owner’s cost coefficient for data value co-creation
I(t)	Data value level
$\beta_{C}$ $\beta_{D}$ $\beta_{O}$	Contractor’s utility coefficient of data value level Designer’s utility coefficient of data value level Owner’s utility coefficient of data value level
δ	Decay coefficient of data value level
R(t)	The total returns of data value co-creation
θ	Utility coefficient of the data value converted into returns
$\xi_{\text{1}}$	Return allocation coefficient for the contractor
$\xi_{\text{2}}$	Return allocation coefficient for the designer
$\xi_{\text{3}}$	Return allocation coefficient for the owner
ρ	Discount rate
$\sigma_{\text{1}}$	Subsidy ratio for the contractor in the decentralized decision-making scenario
$\sigma_{\text{2}}$	Subsidy ratio for the designer in the decentralized decision-making scenario
$\sigma_{\text{3}}$	Subsidy ratio for the owner in the decentralized decision-making scenario
$\varepsilon_{\text{1}}$	Subsidy ratio for the contractor in the centralized decision-making scenario
$\varepsilon_{\text{2}}$	Subsidy ratio for the designer in the centralized decision-making scenario
$\varepsilon_{\text{3}}$	Subsidy ratio for the owner in the centralized decision-making scenario

## 4. Model Solution

### 4.1. Decentralized decision-making scenario

Under the decentralized decision-making scenario, the contractor, designer and owner make decisions independently. It is assumed that there exist continuous and bounded differential profit functions $\overset{N}{\underset{C}{V}}\left(I\right)$, $\overset{N}{\underset{D}{V}}\left(I\right)$ and $\overset{N}{\underset{O}{V}}\left(I\right)$, that satisfy the Hamilton-Jacobi-Bellman (HJB) equation for any I≥0. The continuous-time game can be regarded as the limit form of the discrete-time game when the time interval is infinitely shortened [47]. Then, we have (for the convenience of the solution, the variable t is omitted in the following calculation process):


$$\begin{array}{c} \rho \overset{N}{\underset{C}{V}}\left(I\right)\text{=}\underset{X\geq 0}{max}\left[\xi_{\text{1}}\theta I-\frac{\text{1}}{\text{2}}\alpha_{C}X^{\text{2}}+{\overset{N}{\underset{C}{V}}}^{\prime }\left(I\right)(\beta_{C}X\text{+}\beta_{D}Y+\beta_{O}Z-\delta I)\right] \end{array}$$ (11)

$$\begin{array}{c} \rho \overset{N}{\underset{D}{V}}\left(I\right)\text{=}\underset{Y\geq 0}{max}\left[\xi_{\text{2}}\theta I-\frac{\text{1}}{\text{2}}\alpha_{D}Y^{\text{2}}+{\overset{N}{\underset{D}{V}}}^{\prime }\left(I\right)(\beta_{C}X\text{+}\beta_{D}Y+\beta_{O}Z-\delta I)\right] \end{array}$$ (12)

$$\begin{array}{c} \rho \overset{N}{\underset{O}{V}}\left(I\right)\text{=}\underset{Z\geq 0}{max}\left[\xi_{\text{3}}\theta I-\frac{\text{1}}{\text{2}}\alpha_{O}Z^{\text{2}}+{\overset{N}{\underset{O}{V}}}^{\prime }\left(I\right)(\beta_{C}X\text{+}\beta_{D}Y+\beta_{O}Z-\delta I)\right] \end{array}$$ (13)

Compute the first-order partial derivatives with respect to X, Y and Z for Eqs.(11), (12) and (13), the following functions are obtained:


$$\begin{array}{c} X_{N}=\frac{\beta_{C}{\overset{N}{\underset{C}{V}}}^{\prime }\left(I\right)}{\alpha_{C}} \end{array}$$ (14)

$$\begin{array}{c} Y_{N}=\frac{\beta_{D}{\overset{N}{\underset{D}{V}}}^{\prime }\left(I\right)}{\alpha_{D}} \end{array}$$ (15)

$$\begin{array}{c} Z_{N}=\frac{\beta_{O}\overset{N^{\prime }}{\underset{O}{V}}\left(I\right)}{\alpha_{O}} \end{array}$$ (16)

Substituting Eqs.(14), (15) and (16) into the Eqs.(11), (12) and (13), the following functions are obtained:


$$\begin{array}{c} \rho \overset{N}{\underset{C}{V}}\left(I\right)=\left[\xi_{\text{1}}\theta -\delta \overset{N^{\prime }}{\underset{C}{V}}\left(I\right)\right]I+\frac{{\left[\beta_{C}\overset{N^{\prime }}{\underset{C}{V}}\left(I\right)\right]}^{\text{2}}}{2\alpha_{C}}+\frac{\overset{\text{2}}{\underset{D}{\beta }}\overset{N^{\prime }}{\underset{C}{V}}\left(I\right)\overset{N^{\prime }}{\underset{D}{V}}\left(I\right)}{\alpha_{D}}+\frac{\overset{\text{2}}{\underset{O}{\beta }}\overset{N^{\prime }}{\underset{C}{V}}\left(I\right)\overset{N^{\prime }}{\underset{O}{V}}\left(I\right)}{\alpha_{O}} \end{array}$$ (17)

$$\begin{array}{c} \rho \overset{N}{\underset{D}{V}}\left(I\right)=\left[\xi_{\text{2}}\theta -\delta \overset{N^{\prime }}{\underset{D}{V}}\left(I\right)\right]I+\frac{{\left[\beta_{D}\overset{N^{\prime }}{\underset{D}{V}}\left(I\right)\right]}^{\text{2}}}{2\alpha_{D}}+\frac{\overset{\text{2}}{\underset{C}{\beta }}\overset{N^{\prime }}{\underset{D}{V}}\left(I\right)\overset{N^{\prime }}{\underset{C}{V}}\left(I\right)}{\alpha_{C}}+\frac{\overset{\text{2}}{\underset{O}{\beta }}\overset{N^{\prime }}{\underset{D}{V}}\left(I\right)\overset{N^{\prime }}{\underset{O}{V}}\left(I\right)}{\alpha_{O}} \end{array}$$ (18)

$$\begin{array}{c} \rho \overset{N}{\underset{O}{V}}\left(I\right)=\left[\xi_{\text{3}}\theta -\delta \overset{N^{\prime }}{\underset{O}{V}}\left(I\right)\right]I+\frac{{\left[\beta_{O}\overset{N^{\prime }}{\underset{O}{V}}\left(I\right)\right]}^{\text{2}}}{2\alpha_{O}}+\frac{\overset{\text{2}}{\underset{C}{\beta }}\overset{N^{\prime }}{\underset{O}{V}}\left(I\right)\overset{N^{\prime }}{\underset{C}{V}}\left(I\right)}{\alpha_{C}}+\frac{\overset{\text{2}}{\underset{D}{\beta }}\overset{N^{\prime }}{\underset{O}{V}}\left(I\right)\overset{N^{\prime }}{\underset{D}{V}}\left(I\right)}{\alpha_{D}} \end{array}$$ (19)

Based on the structural characteristics of the differential Eqs. (17), (18) and (19), the linear profit function with respect to I is the solution to the HJB equation [48]. Therefore, it is assumed that the expressions of $\overset{N}{\underset{C}{V}}\left(I\right)$, $\overset{N}{\underset{D}{V}}\left(I\right)$ and $\overset{N}{\underset{O}{V}}\left(I\right)$ are as:


$$\begin{array}{c} \overset{N}{\underset{C}{V}}\left(I\right)=a_{\text{1}}I+b_{\text{1}} \end{array}$$ (20)

$$\begin{array}{c} \overset{N}{\underset{D}{V}}\left(I\right)=a_{\text{2}}I+b_{\text{2}} \end{array}$$ (21)

$$\begin{array}{c} \overset{N}{\underset{O}{V}}\left(I\right)=a_{\text{3}}I+b_{\text{3}} \end{array}$$ (22)

where $a_{\text{1}}$, $a_{\text{2}}$, $a_{\text{3}}$, $b_{\text{1}}$, $b_{\text{2}}$, $b_{\text{3}}$ are all constants.

Substituting the expressions of $\overset{N}{\underset{C}{V}}\left(I\right)$, $\overset{N}{\underset{D}{V}}\left(I\right)$, $\overset{N}{\underset{O}{V}}\left(I\right)$ and their derivatives into Eqs.(17), (18) and (19), the following functions can be obtained:


$$\begin{array}{c} a_{\text{1}}=\frac{\xi_{\text{1}}\theta }{\rho +\delta } \end{array}$$ (23)

$$\begin{array}{c} a_{\text{2}}=\frac{\xi_{\text{2}}\theta }{\rho +\delta } \end{array}$$ (24)

$$\begin{array}{c} a_{\text{3}}=\frac{\xi_{\text{3}}\theta }{\rho +\delta } \end{array}$$ (25)

$$\begin{array}{c} b_{\text{1}}=\frac{{\left(\xi_{\text{1}}\theta \beta_{C}\right)}^{\text{2}}}{2\rho \alpha_{C}{\left(\rho +\delta \right)}^{\text{2}}}+\frac{\xi_{\text{1}}\xi_{\text{2}}{\left(\theta \beta_{D}\right)}^{\text{2}}}{\rho \alpha_{D}{\left(\rho +\delta \right)}^{\text{2}}}+\frac{\xi_{\text{1}}\xi_{\text{3}}{\left(\theta \beta_{O}\right)}^{\text{2}}}{\rho \alpha_{O}{\left(\rho +\delta \right)}^{\text{2}}} \end{array}$$ (26)

$$\begin{array}{c} b_{\text{2}}=\frac{{\left(\xi_{\text{2}}\theta \beta_{D}\right)}^{\text{2}}}{2\rho \alpha_{D}{\left(\rho +\delta \right)}^{\text{2}}}+\frac{\xi_{\text{2}}\xi_{\text{1}}{\left(\theta \beta_{C}\right)}^{\text{2}}}{\rho \alpha_{C}{\left(\rho +\delta \right)}^{\text{2}}}+\frac{\xi_{\text{2}}\xi_{\text{3}}{\left(\theta \beta_{O}\right)}^{\text{2}}}{\rho \alpha_{O}{\left(\rho +\delta \right)}^{\text{2}}} \end{array}$$ (27)

$$\begin{array}{c} b_{\text{3}}=\frac{{\left(\xi_{\text{3}}\theta \beta_{O}\right)}^{\text{2}}}{2\rho \alpha_{O}{\left(\rho +\delta \right)}^{\text{2}}}+\frac{\xi_{\text{3}}\xi_{\text{1}}{\left(\theta \beta_{C}\right)}^{\text{2}}}{\rho \alpha_{C}{\left(\rho +\delta \right)}^{\text{2}}}+\frac{\xi_{\text{3}}\xi_{\text{2}}{\left(\theta \beta_{D}\right)}^{\text{2}}}{\rho \alpha_{D}{\left(\rho +\delta \right)}^{\text{2}}} \end{array}$$ (28)

Substituting Eqs.(23), (24), (25), (26), (27) and (28) into Eqs.(20), (21) and (22). Then, the optimal profit expressions of the contractor, designer and owner under the decentralized decision-making scenario are obtained as:


$$\begin{array}{c} \overset{N^{*}}{\underset{C}{V}}\left(I\right)=\frac{\xi_{\text{1}}\theta }{\rho +\delta }I+\frac{{\left(\xi_{\text{1}}\theta \beta_{C}\right)}^{\text{2}}}{2\rho \alpha_{C}{\left(\rho +\delta \right)}^{\text{2}}}+\frac{\xi_{\text{1}}\xi_{\text{2}}{\left(\theta \beta_{D}\right)}^{\text{2}}}{\rho \alpha_{D}{\left(\rho +\delta \right)}^{\text{2}}}+\frac{\xi_{\text{1}}\xi_{\text{3}}{\left(\theta \beta_{O}\right)}^{\text{2}}}{\rho \alpha_{O}{\left(\rho +\delta \right)}^{\text{2}}} \end{array}$$ (29)

$$\begin{array}{c} \overset{N^{*}}{\underset{D}{V}}\left(I\right)=\frac{\xi_{\text{2}}\theta }{\rho +\delta }I+\frac{{\left(\xi_{\text{2}}\theta \beta_{D}\right)}^{\text{2}}}{2\rho \alpha_{D}{\left(\rho +\delta \right)}^{\text{2}}}+\frac{\xi_{\text{2}}\xi_{\text{1}}{\left(\theta \beta_{C}\right)}^{\text{2}}}{\rho \alpha_{C}{\left(\rho +\delta \right)}^{\text{2}}}+\frac{\xi_{\text{2}}\xi_{\text{3}}{\left(\theta \beta_{O}\right)}^{\text{2}}}{\rho \alpha_{O}{\left(\rho +\delta \right)}^{\text{2}}} \end{array}$$ (30)

$$\begin{array}{c} \overset{N^{*}}{\underset{O}{V}}\left(I\right)=\frac{\xi_{\text{3}}\theta }{\rho +\delta }I+\frac{{\left(\xi_{\text{3}}\theta \beta_{O}\right)}^{\text{2}}}{2\rho \alpha_{O}{\left(\rho +\delta \right)}^{\text{2}}}+\frac{\xi_{\text{3}}\xi_{\text{1}}{\left(\theta \beta_{C}\right)}^{\text{2}}}{\rho \alpha_{C}{\left(\rho +\delta \right)}^{\text{2}}}+\frac{\xi_{\text{3}}\xi_{\text{2}}{\left(\theta \beta_{D}\right)}^{\text{2}}}{\rho \alpha_{D}{\left(\rho +\delta \right)}^{\text{2}}} \end{array}$$ (31)

Substitute Eqs.(29), (30) and (31) into Eqs.(14), (15) and (16). The equilibrium solutions for the data value co-creation effort levels of the contractor, designer and owner can be obtained as:


$$\begin{array}{c} {X_{N}}^{*}=\frac{\xi_{\text{1}}\theta \beta_{C}}{\alpha_{C}\left(\rho +\delta \right)} \end{array}$$ (32)

$$\begin{array}{c} {Y_{N}}^{*}=\frac{\xi_{\text{2}}\theta \beta_{D}}{\alpha_{D}\left(\rho +\delta \right)} \end{array}$$ (33)

$$\begin{array}{c} {Z_{N}}^{*}=\frac{\xi_{\text{3}}\theta \beta_{O}}{\alpha_{O}\left(\rho +\delta \right)} \end{array}$$ (34)

Substitute Eqs. (32), (33), and (34) into the right-hand part of Eq.(1), then set it equal to zero. The optimal trajectory $\overset{*}{\underset{N}{I}}\left(t\right)$ of the data value level under the decentralized decision-making scenario can be obtained as:


$$\begin{array}{c} \overset{*}{\underset{N}{I}}\left(t\right)={\eta_{N}}^{*}+e^{-\delta t}(I_{\text{0}}-{\eta_{N}}^{*}) \end{array}$$ (35)

where, ${\eta_{N}}^{*}\text{=}\frac{\overset{\text{2}}{\underset{C}{\beta }}\xi_{\text{1}}\theta }{\delta \alpha_{C}\left(\rho +\delta \right)}+\frac{\overset{\text{2}}{\underset{D}{\beta }}\xi_{\text{2}}\theta }{\delta \alpha_{D}\left(\rho +\delta \right)}+\frac{\overset{\text{2}}{\underset{O}{\beta }}\xi_{\text{3}}\theta }{\delta \alpha_{O}\left(\rho +\delta \right)}$.

### 4.2. Decentralized decision-making scenario with government subsidies

Under the decentralized decision-making scenario with government subsidies, the objective profit functions of the contractor, designer, and owner $\overset{S}{\underset{C}{V}}\left(I\right)$, $\overset{S}{\underset{D}{V}}\left(I\right)$ and $\overset{S}{\underset{O}{V}}\left(I\right)$ satisfy the HJB equation for any I≥0, specifically:


$$\begin{array}{c} \rho \overset{S}{\underset{C}{V}}\left(I\right)\text{\textasciitilde{}= }\underset{X\geq 0}{max}\left[\xi_{\text{1}}\theta I-\frac{\text{1}}{\text{2}}\alpha_{C}X^{\text{2}}\text{+}\frac{\text{1}}{\text{2}}\sigma_{\text{1}}\alpha_{C}X^{\text{2}}+{\overset{S}{\underset{C}{V}}}^{\prime }\left(I\right)(\beta_{C}X\text{+}\beta_{D}Y+\beta_{O}Z-\delta I)\right] \end{array}$$ (36)

$$\begin{array}{c} \rho \overset{S}{\underset{D}{V}}\left(I\right)\text{\textasciitilde{}=\textasciitilde{}}\underset{Y\geq 0}{max}\left[\xi_{\text{2}}\theta I-\frac{\text{1}}{\text{2}}\alpha_{D}Y^{\text{2}}\text{+}\frac{\text{1}}{\text{2}}\sigma_{\text{2}}\alpha_{D}Y^{\text{2}}\text{+}{\overset{S}{\underset{D}{V}}}^{\prime }\left(I\right)(\beta_{C}X\text{+}\beta_{D}Y+\beta_{O}Z-\delta I)\right] \end{array}$$ (37)

$$\begin{array}{c} \rho \overset{S}{\underset{O}{V}}\left(I\right)\text{\textasciitilde{}=\textasciitilde{}}\underset{Z\geq 0}{max}\left[\xi_{\text{3}}\theta I-\frac{\text{1}}{\text{2}}\alpha_{O}Z^{\text{2}}\text{+}\frac{\text{1}}{\text{2}}\sigma_{\text{3}}\alpha_{O}Z^{\text{2}}\text{+}{\overset{S}{\underset{O}{V}}}^{\prime }\left(I\right)(\beta_{C}X\text{+}\beta_{D}Y+\beta_{O}Z-\delta I)\right] \end{array}$$ (38)

Based on the HJB approach, the optimal profit and optimal effort levels of the contractor, designer, and owner under decentralized decision-making with government subsidies can be obtained as follows:


$$\begin{array}{c} \overset{S^{*}}{\underset{C}{V}}\left(I\right)=\frac{\xi_{\text{1}}\theta }{\rho +\delta }I+\frac{{\left(\xi_{\text{1}}\theta \beta_{C}\right)}^{\text{2}}}{2\rho \alpha_{C}{\left(\rho +\delta \right)}^{\text{2}}(1-\sigma_{\text{1}})}+\frac{\xi_{\text{1}}\xi_{\text{2}}{\left(\theta \beta_{D}\right)}^{\text{2}}}{\rho \alpha_{D}{\left(\rho +\delta \right)}^{\text{2}}(1-\sigma_{\text{2}})}+\frac{\xi_{\text{1}}\xi_{\text{3}}{\left(\theta \beta_{O}\right)}^{\text{2}}}{\rho \alpha_{O}{\left(\rho +\delta \right)}^{\text{2}}(1-\sigma_{\text{3}})} \end{array}$$ (39)

$$\begin{array}{c} \overset{S^{*}}{\underset{D}{V}}\left(I\right)=\frac{\xi_{\text{2}}\theta }{\rho +\delta }I+\frac{{\left(\xi_{\text{2}}\theta \beta_{D}\right)}^{\text{2}}}{2\rho \alpha_{D}{\left(\rho +\delta \right)}^{\text{2}}(1-\sigma_{\text{2}})}+\frac{\xi_{\text{2}}\xi_{\text{1}}{\left(\theta \beta_{C}\right)}^{\text{2}}}{\rho \alpha_{C}{\left(\rho +\delta \right)}^{\text{2}}(1-\sigma_{\text{1}})}+\frac{\xi_{\text{2}}\xi_{\text{3}}{\left(\theta \beta_{O}\right)}^{\text{2}}}{\rho \alpha_{O}{\left(\rho +\delta \right)}^{\text{2}}(1-\sigma_{\text{3}})} \end{array}$$ (40)

$$\begin{array}{c} \overset{S^{*}}{\underset{O}{V}}\left(I\right)=\frac{\xi_{\text{3}}\theta }{\rho +\delta }I+\frac{{\left(\xi_{\text{3}}\theta \beta_{O}\right)}^{\text{2}}}{2\rho \alpha_{O}{\left(\rho +\delta \right)}^{\text{2}}(1-\sigma_{\text{3}})}+\frac{\xi_{\text{3}}\xi_{\text{1}}{\left(\theta \beta_{C}\right)}^{\text{2}}}{\rho \alpha_{C}{\left(\rho +\delta \right)}^{\text{2}}(1-\sigma_{\text{1}})}+\frac{\xi_{\text{3}}\xi_{\text{2}}{\left(\theta \beta_{D}\right)}^{\text{2}}}{\rho \alpha_{D}{\left(\rho +\delta \right)}^{\text{2}}(1-\sigma_{\text{2}})} \end{array}$$ (41)

$$\begin{array}{c} {X_{S}}^{*}=\frac{\xi_{\text{1}}\theta \beta_{C}}{\alpha_{C}\left(\rho +\delta \right)(1-\sigma_{\text{1}})} \end{array}$$ (42)

$$\begin{array}{c} {Y_{S}}^{*}=\frac{\xi_{\text{2}}\theta \beta_{D}}{\alpha_{D}\left(\rho +\delta \right)(1-\sigma_{\text{2}})} \end{array}$$ (43)

$$\begin{array}{c} {Z_{S}}^{*}=\frac{\xi_{\text{3}}\theta \beta_{O}}{\alpha_{O}\left(\rho +\delta \right)(1-\sigma_{\text{3}})} \end{array}$$ (44)

The optimal trajectory $\overset{*}{\underset{S}{I}}\left(t\right)$ of the data value level can be obtained as:


$$\begin{array}{c} \overset{*}{\underset{S}{I}}\left(t\right)={\eta_{S}}^{*}+e^{-\delta t}(I_{\text{0}}-{\eta_{S}}^{*}) \end{array}$$ (45)

where, ${\eta_{S}}^{*}=\frac{\overset{\text{2}}{\underset{C}{\beta }}\xi_{\text{1}}\theta }{\delta \alpha_{C}\left(\rho +\delta \right)(1-\sigma_{\text{1}})}+\frac{\overset{\text{2}}{\underset{D}{\beta }}\xi_{\text{2}}\theta }{\delta \alpha_{D}\left(\rho +\delta \right)(1-\sigma_{\text{2}})}+\frac{\overset{\text{2}}{\underset{O}{\beta }}\xi_{\text{3}}\theta }{\delta \alpha_{O}\left(\rho +\delta \right)(1-\sigma_{\text{3}})}$.

### 4.3. Centralized decision-making scenario

In the centralized decision-making scenario, the optimal value function $V_{A}\left(I\right)$ of the contractor, designer and owner satisfies the HJB equation for any I≥0, specifically:


$$\begin{array}{c} \rho V_{A}\left(I\right)\text{=}\underset{X\geq 0,Y\geq 0,Z\geq 0}{max}\left[\theta I-\frac{\text{1}}{\text{2}}\alpha_{C}X^{\text{2}}-\frac{\text{1}}{\text{2}}\alpha_{D}Y^{\text{2}}-\frac{\text{1}}{\text{2}}\alpha_{O}Z^{\text{2}}+{V_{A}}^{\prime }\left(I\right)\left(\beta_{C}X\text{+}\beta_{D}Y+\beta_{O}Z-\delta I\right)\right] \end{array}$$ (46)

Based on the HJB approach, the optimal profit and effort levels functions of the contractor, designer, and owner for data value co-creation under centralized decision-making can be obtained as follows:


$$\begin{array}{c} \overset{*}{\underset{A}{V}}\left(I\right)=\frac{\theta }{\rho +\delta }I+\frac{{\left(\theta \beta_{C}\right)}^{\text{2}}}{2\rho \alpha_{C}{\left(\rho +\delta \right)}^{\text{2}}}+\frac{{\left(\theta \beta_{D}\right)}^{\text{2}}}{2\rho \alpha_{D}{\left(\rho +\delta \right)}^{\text{2}}}+\frac{{\left(\theta \beta_{O}\right)}^{\text{2}}}{2\rho \alpha_{O}{\left(\rho +\delta \right)}^{\text{2}}} \end{array}$$ (47)

$$\begin{array}{c} {X_{A}}^{*}=\frac{\theta \beta_{C}}{\alpha_{C}\left(\rho +\delta \right)} \end{array}$$ (48)

$$\begin{array}{c} {Y_{A}}^{*}=\frac{\theta \beta_{D}}{\alpha_{D}\left(\rho +\delta \right)} \end{array}$$ (49)

$$\begin{array}{c} {Z_{A}}^{*}=\frac{\theta \beta_{O}}{\alpha_{O}\left(\rho +\delta \right)} \end{array}$$ (50)

The optimal trajectory $\overset{*}{\underset{A}{I}}\left(t\right)$ of the data value level under the centralized decision-making scenario can be obtained as:


$$\begin{array}{c} \overset{*}{\underset{A}{I}}\left(t\right)={\eta_{A}}^{*}+e^{-\delta t}(I_{\text{0}}-{\eta_{A}}^{*}) \end{array}$$ (51)

where, ${\eta_{A}}^{*}=\frac{\overset{\text{2}}{\underset{C}{\beta }}\theta }{\delta \alpha_{C}\left(\rho +\delta \right)}+\frac{\overset{\text{2}}{\underset{D}{\beta }}\theta }{\delta \alpha_{D}\left(\rho +\delta \right)}+\frac{\overset{\text{2}}{\underset{O}{\beta }}\theta }{\delta \alpha_{O}\left(\rho +\delta \right)}$.

### 4.4. Centralized decision-making scenario with government subsidies

In the centralized decision-making scenario with government subsidies, the optimal value function $V_{T}\left(I\right)$ of the contractor, designer and owner satisfies the HJB equation for any I≥0, specifically:


$$\begin{array}{c} \begin{array}{c} \rho V_{T}\left(I\right)\text{=}\underset{X\geq 0,Y\geq 0,Z\geq 0}{max}[\theta I-\frac{\text{1}}{\text{2}}\alpha_{C}X^{\text{2}}-\frac{\text{1}}{\text{2}}\alpha_{D}Y^{\text{2}}-\frac{\text{1}}{\text{2}}\alpha_{O}Z^{\text{2}}+\frac{\text{1}}{\text{2}}\varepsilon_{\text{1}}\alpha_{C}X^{\text{2}}+\frac{\text{1}}{\text{2}}\varepsilon_{\text{2}}\alpha_{D}Y^{\text{2}}+\frac{\text{1}}{\text{2}}\varepsilon_{\text{3}}\alpha_{O}Z^{\text{2}}\\ +{V_{T}}^{\prime }\left(I\right)\left(\beta_{C}X\text{+}\beta_{D}Y+\beta_{O}Z-\delta I\right)] \end{array} \end{array}$$ (52)

Based on the HJB approach, the optimal profit and optimal effort levels functions of the contractor, designer, and owner in data value co-creation can be obtained as follows:


$$\begin{array}{c} {V_{T}}^{*}\left(I\right)=\frac{\theta }{\rho +\delta }I+\frac{{\left(\theta \beta_{C}\right)}^{\text{2}}}{2\rho \alpha_{C}{\left(\rho +\delta \right)}^{\text{2}}(1-\varepsilon_{\text{1}})}+\frac{{\left(\theta \beta_{D}\right)}^{\text{2}}}{2\rho \alpha_{D}{\left(\rho +\delta \right)}^{\text{2}}(1-\varepsilon_{\text{2}})}+\frac{{\left(\theta \beta_{O}\right)}^{\text{2}}}{2\rho \alpha_{O}{\left(\rho +\delta \right)}^{\text{2}}(1-\varepsilon_{\text{3}})} \end{array}$$ (53)

$$\begin{array}{c} {X_{T}}^{*}=\frac{\theta \beta_{C}}{\alpha_{C}\left(\rho +\delta \right)(1-\varepsilon_{\text{1}})} \end{array}$$ (54)

$$\begin{array}{c} {Y_{T}}^{*}=\frac{\theta \beta_{D}}{\alpha_{D}\left(\rho +\delta \right)(1-\varepsilon_{\text{2}})} \end{array}$$ (55)

$$\begin{array}{c} {Z_{T}}^{*}=\frac{\theta \beta_{O}}{\alpha_{O}\left(\rho +\delta \right)(1-\varepsilon_{\text{3}})} \end{array}$$ (56)

The optimal trajectory $\overset{*}{\underset{T}{I}}\left(t\right)$ of the data value level can be obtained as:


$$\begin{array}{c} \overset{*}{\underset{T}{I}}\left(t\right)={\eta_{T}}^{*}+e^{-\delta t}(I_{\text{0}}-{\eta_{T}}^{*}) \end{array}$$ (57)

where, ${\eta_{T}}^{*}=\frac{\overset{\text{2}}{\underset{C}{\beta }}\theta }{\delta \alpha_{C}\left(\rho +\delta \right)(1-\varepsilon_{\text{1}})}+\frac{\overset{\text{2}}{\underset{D}{\beta }}\theta }{\delta \alpha_{D}\left(\rho +\delta \right)(1-\varepsilon_{\text{2}})}+\frac{\overset{\text{2}}{\underset{O}{\beta }}\theta }{\delta \alpha_{O}\left(\rho +\delta \right)(1-\varepsilon_{\text{3}})}$.

## 5. Simulation analysis

From the above analysis, the optimal effort levels and optimal benefits of the contractor, designer and owner can be derived under four scenarios. This section employs simulation to analyze the equilibrium strategies of participants in data value co-creation and the impact of key factors on the outcomes of data value co-creation.

### 5.1. Parameter setting

Based on the above assumptions, and following the parameter-setting principles in the literature [42,49] while considering the actual situation, the initial parameter values are set as shown in Table 2. In addition, to enhance the reliability of the research findings, this study conducts robustness analysis to examine the stability of the model results.

**Table 2:** Initial parameter value setting.

Parameter	Numerical value	Parameter	Numerical value	Parameter	Numerical value
ρ	0.07	$\xi_{\text{1}}$	0.3	$\sigma_{\text{1}}$	0.3
δ	0.2	$\xi_{\text{2}}$	0.25	$\sigma_{\text{2}}$	0.3
$\beta_{C}$	0.5	$\xi_{\text{3}}$	0.45	$\sigma_{\text{3}}$	0.4
$\beta_{D}$	0.4	$\alpha_{C}$	0.8	$\varepsilon_{\text{1}}$	0.5
$\beta_{O}$	0.8	$\alpha_{D}$	0.7	$\varepsilon_{\text{2}}$	0.5
$I_{\text{0}}$	2	$\alpha_{O}$	0.9	$\varepsilon_{\text{3}}$	0.6
θ	0.8				

### 5.2. The law of data value level changing with time

**Fig 2:**
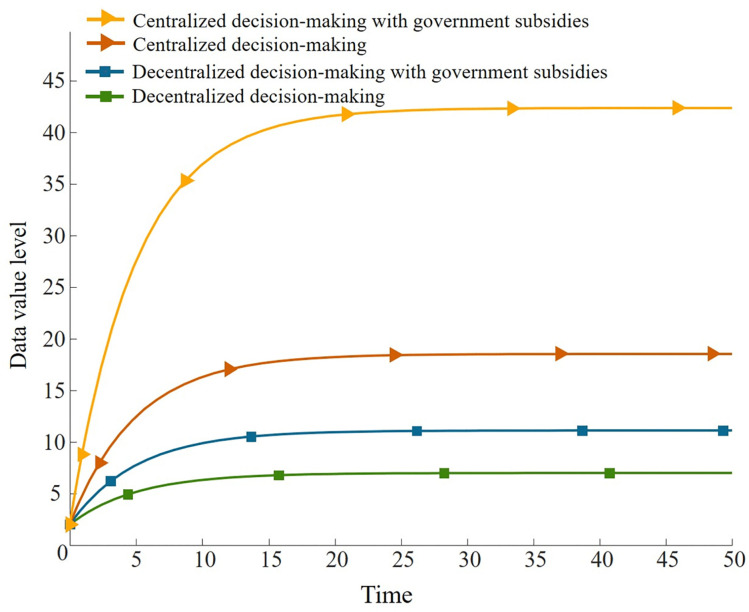
Data value level under different decision-making scenarios.

Based on the parameters in Table 2, simulation analysis is conducted to examine the evolution of data value level over time under the four decision-making scenarios, as shown in Fig 2.

It can be seen from Fig 2 that, under the four decision-making scenarios, the data value level shows an evolutionary characteristic of rapid growth in the early stage followed by stabilization, but there are significant differences. The data value level under the centralized decision-making scenario is higher than that under the decentralized decision-making scenario, indicating that a unified coordination mechanism can improve data integration efficiency and is more conducive to realizing data value co-creation. After introducing government subsidies, the data value level further increases. The centralized decision-making scenario with government subsidies has the fastest growth rate and stabilizes at the highest level. This indicates that government subsidies, as an incentive measure, can encourage all subjects to participate in the co-creation of data value and rapidly improve the level of data value. Therefore, centralized decision-making and government incentives are more conducive to realizing data value co-creation.

### 5.3. The variation law of data value co-creation returns over time

**Fig 3:**
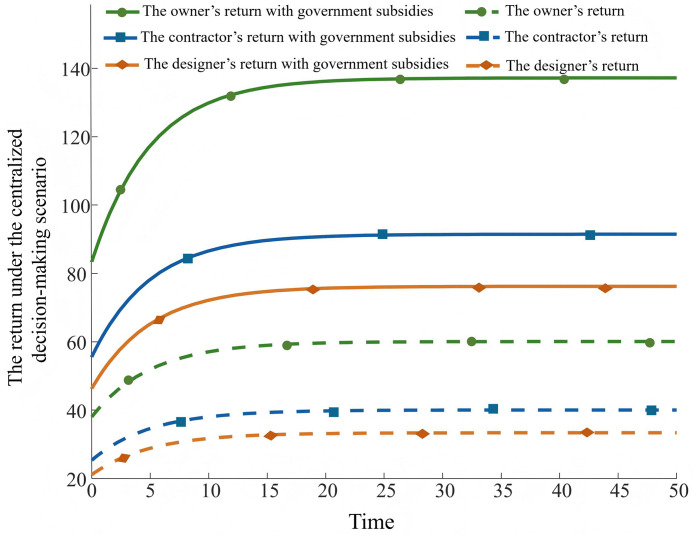
Comparison of returns among contractor, designer and owner under the centralized decision-making scenario.

**Fig 4:**
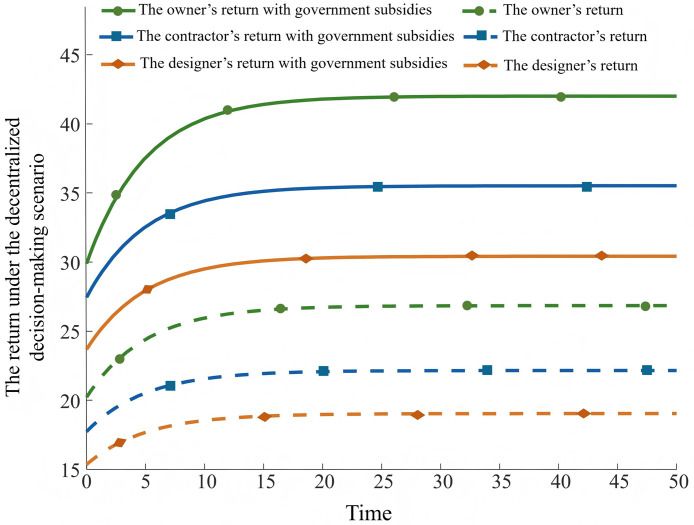
Comparison of returns among contractor, designer and owner under the decentralized decision-making scenario.

**Fig 5:**
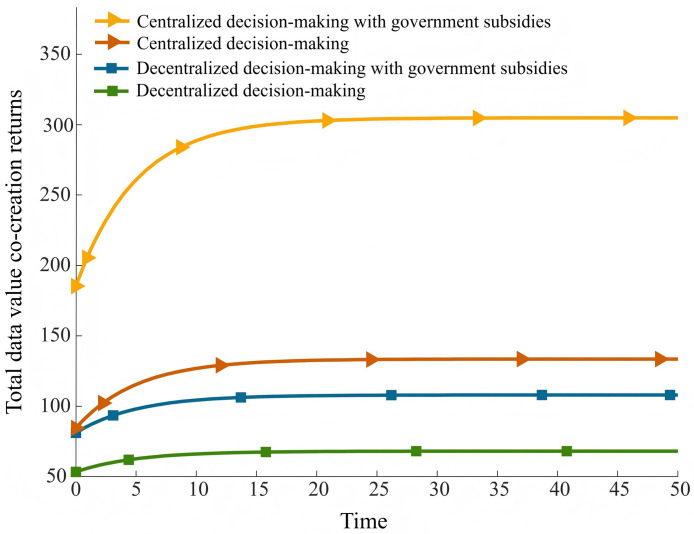
Variations in total data value co-creation returns under different scenarios.

Through simulation analysis, the changes in the returns of contractor, designer, and owner under centralized and decentralized decision-making scenarios are obtained as shown in Figs 3–4. And the change in total returns under the four decision-making scenarios is obtained as shown in Fig 5.

It can be seen from Figs 3–4 that the optimal returns of contractor, designer and owner under both centralized and decentralized decision-making scenarios first increase and then tend to stabilize. The optimal returns of contractor, designer and owner with government subsidies are all higher than those without government subsidies, indicating that government subsidies effectively convert external incentive policies into participants’ returns and promote the growth of participants’ data value co-creation returns.

From Fig 5, the total returns under centralized decision-making are higher than those under decentralized decision-making, and the total returns are the highest when centralized decision-making is combined with government subsidies. This indicates that centralized decision-making improves multi-party collaboration efficiency, while the subsidy mechanism further enhances the investment of each party, thereby increasing the overall return of data value co-creation.

### 5.4. Sensitivity analysis of government subsidy ratio

**Fig 6:**
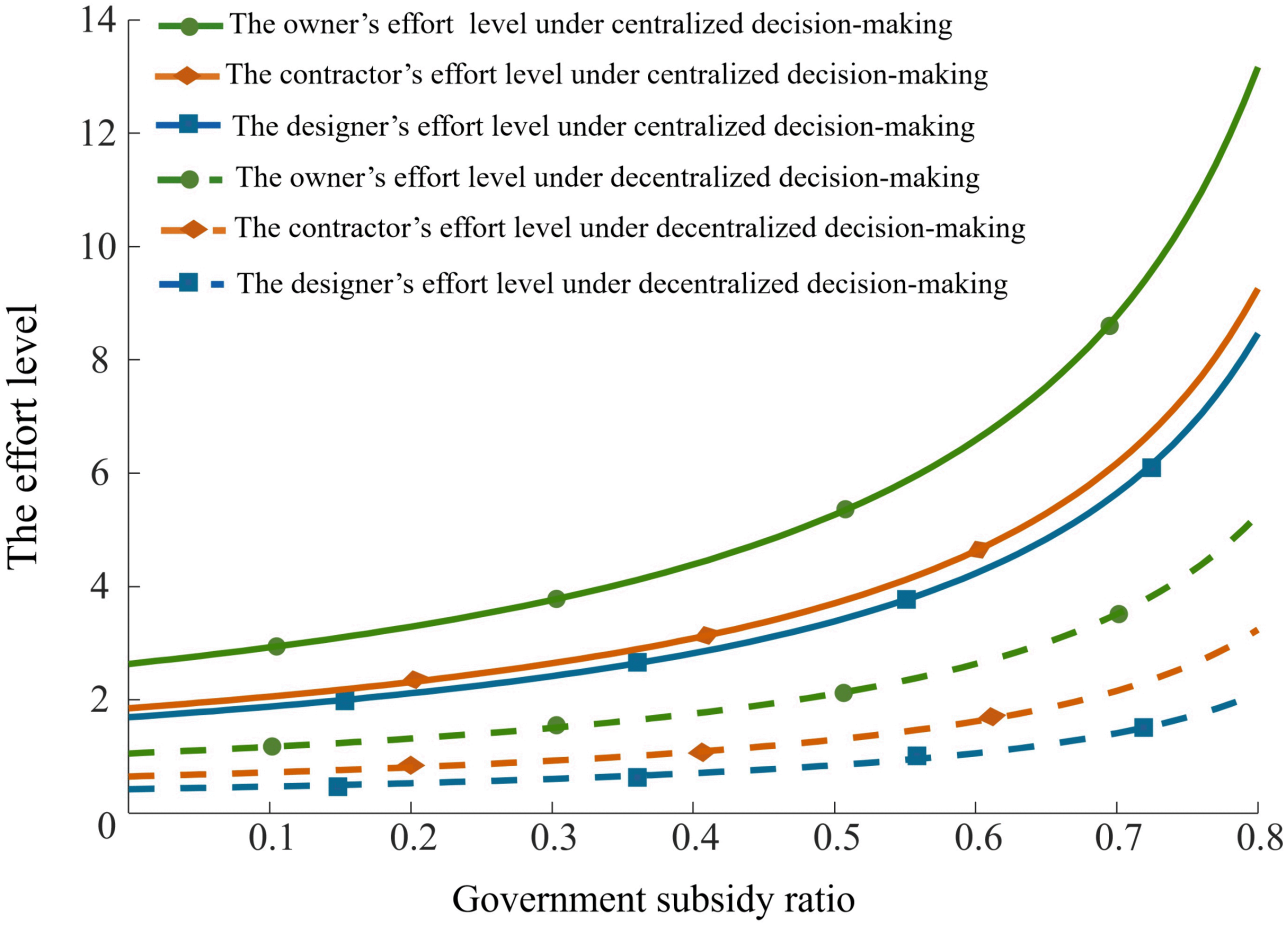
Impact of government subsidy ratio on the effort levels of contractor, designer and owner.

**Fig 7:**
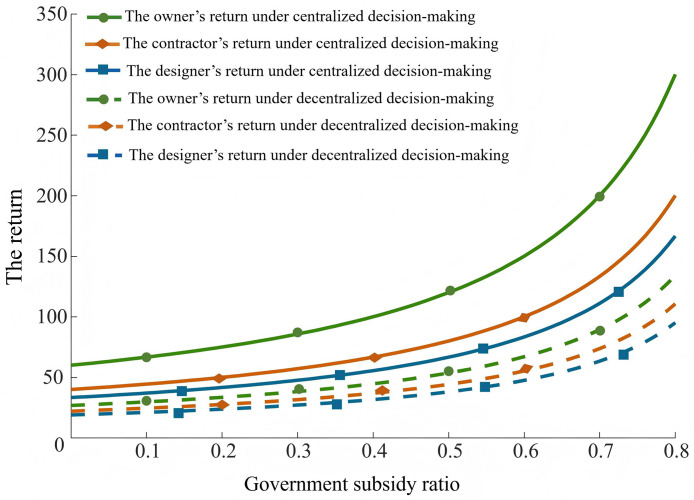
Impact of government subsidy ratio on the returns of contractor, designer and owner.

Similarly, the effects of government subsidies on the effort levels and returns of the contractor, designer, and owner are shown in Figs 6–7.

It can be seen from Figs 6–7 that, with the increase in the government subsidy coefficient, the effort levels and returns of the contractor, designer and owner exhibit an upward trend. The improvements are more pronounced under centralized decision-making. This indicates that government subsidies can effectively reduce the costs of participating in data value co-creation, thereby enhancing the willingness of each participant to invest. Meanwhile, a unified coordination mechanism amplifies the incentive effect of subsidies, facilitating the transformation of policy incentives into actual inputs and improving the overall returns. Therefore, governments should actively promote collaboration among project participants to further enhance the effectiveness of policy incentives for data value co-creation.

### 5.5. Impact of cost coefficients on the effort levels and returns of all parties

**Fig 8:**
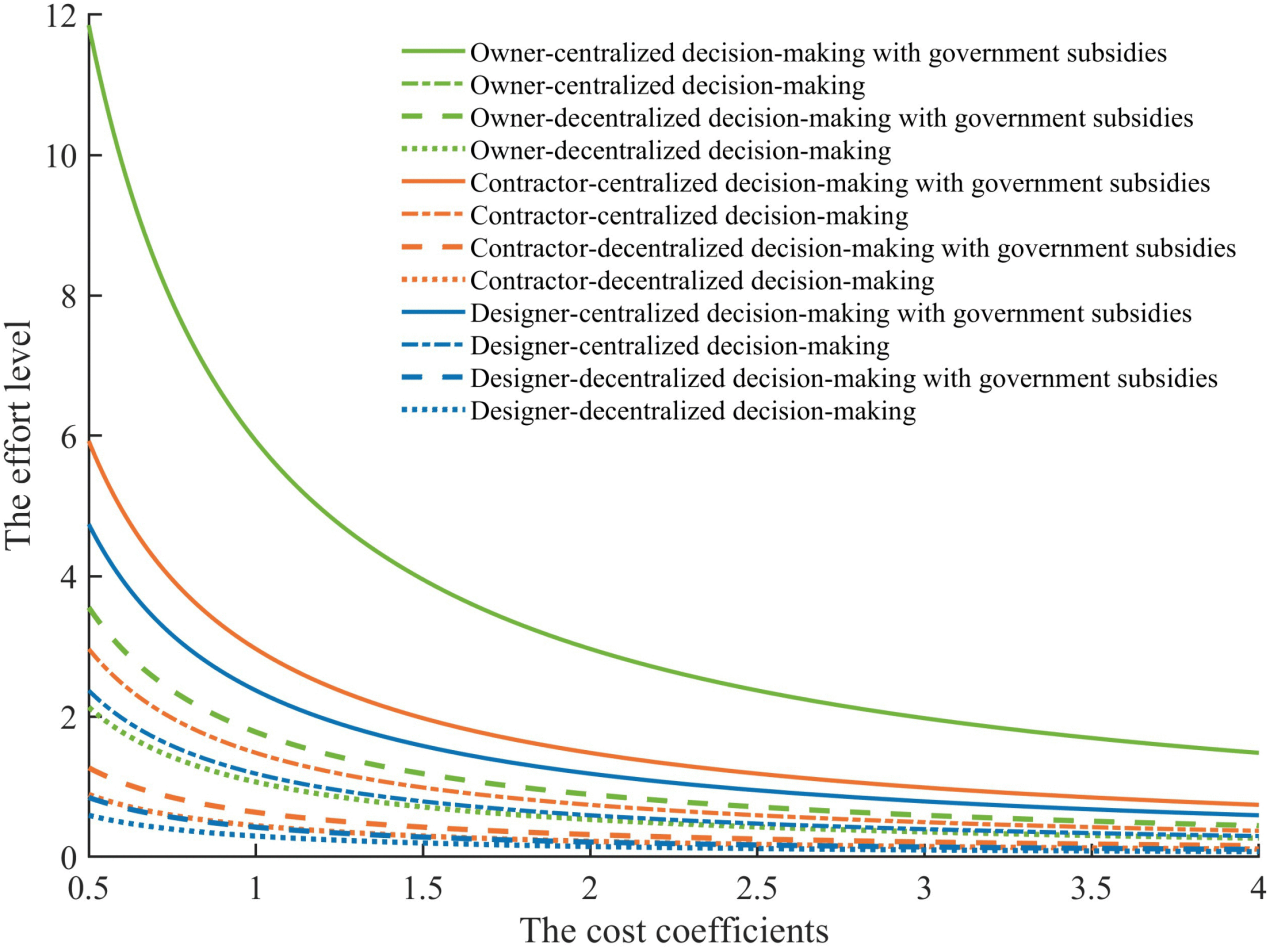
Impact of cost coefficients of contractor, designer and owner on their effort levels.

**Fig 9:**
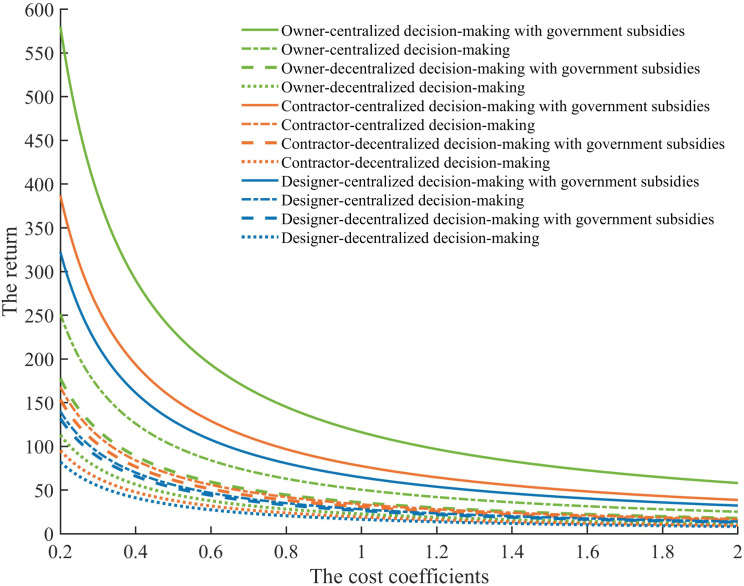
Impact of cost coefficients of contractor, designer and owner on their returns.

The effects of the cost coefficients of contractor, designer, and owner on their effort levels and returns are shown in Figs 8–9.

It can be seen from Figs 8–9 that, with the increase in the cost coefficients, the effort levels and returns of all parties show a downward trend. This suggests that higher costs significantly inhibit collaboration among the parties. When data value co-creation costs are high, the parties reduce their willingness to invest, thereby weakening the level of data value co-creation.

### 5.6. Impact of utility coefficients on the effort levels and returns of all parties

**Fig 10:**
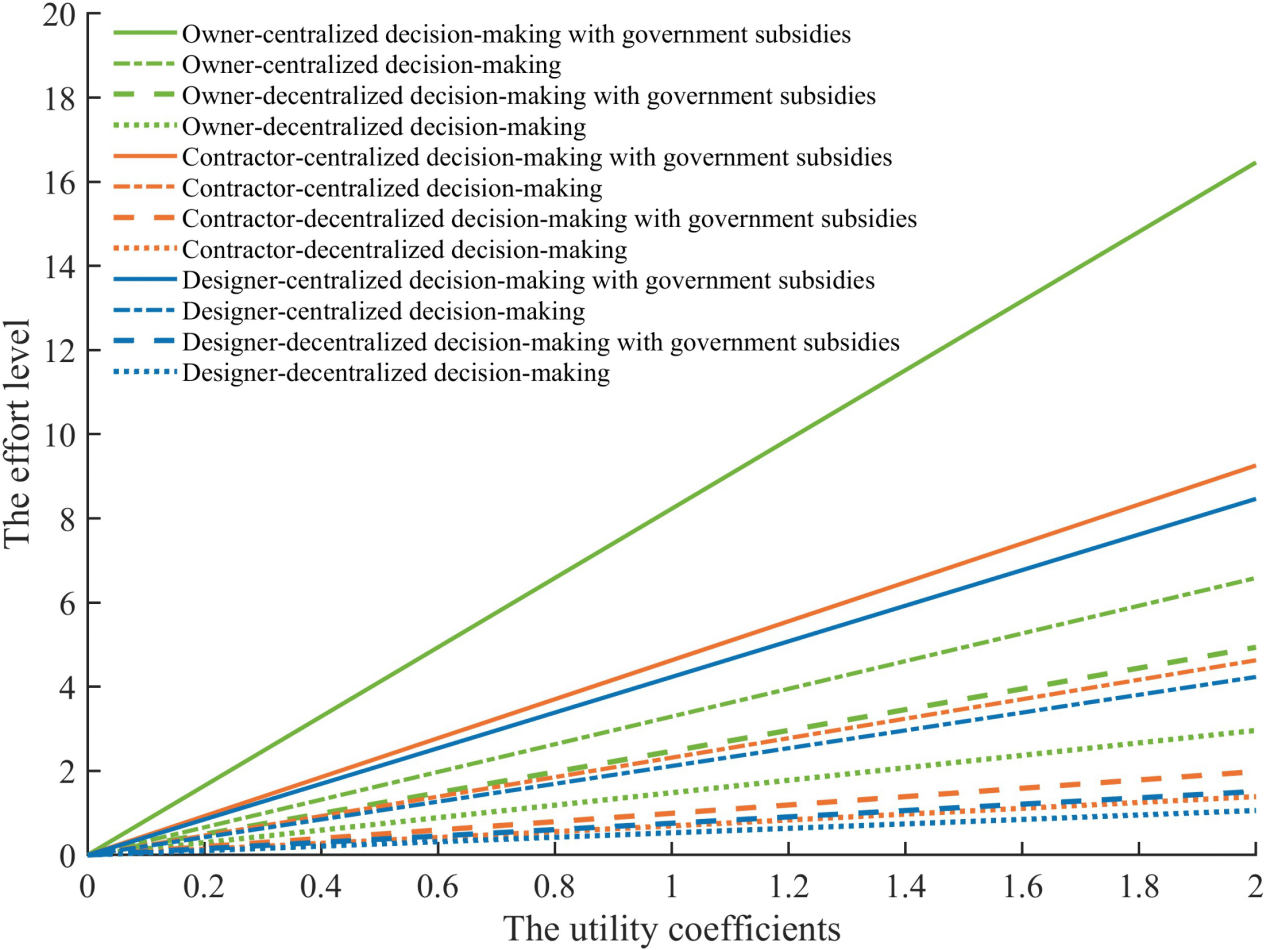
Impact of utility coefficients of contractor, designer and owner on their effort levels.

**Fig 11:**
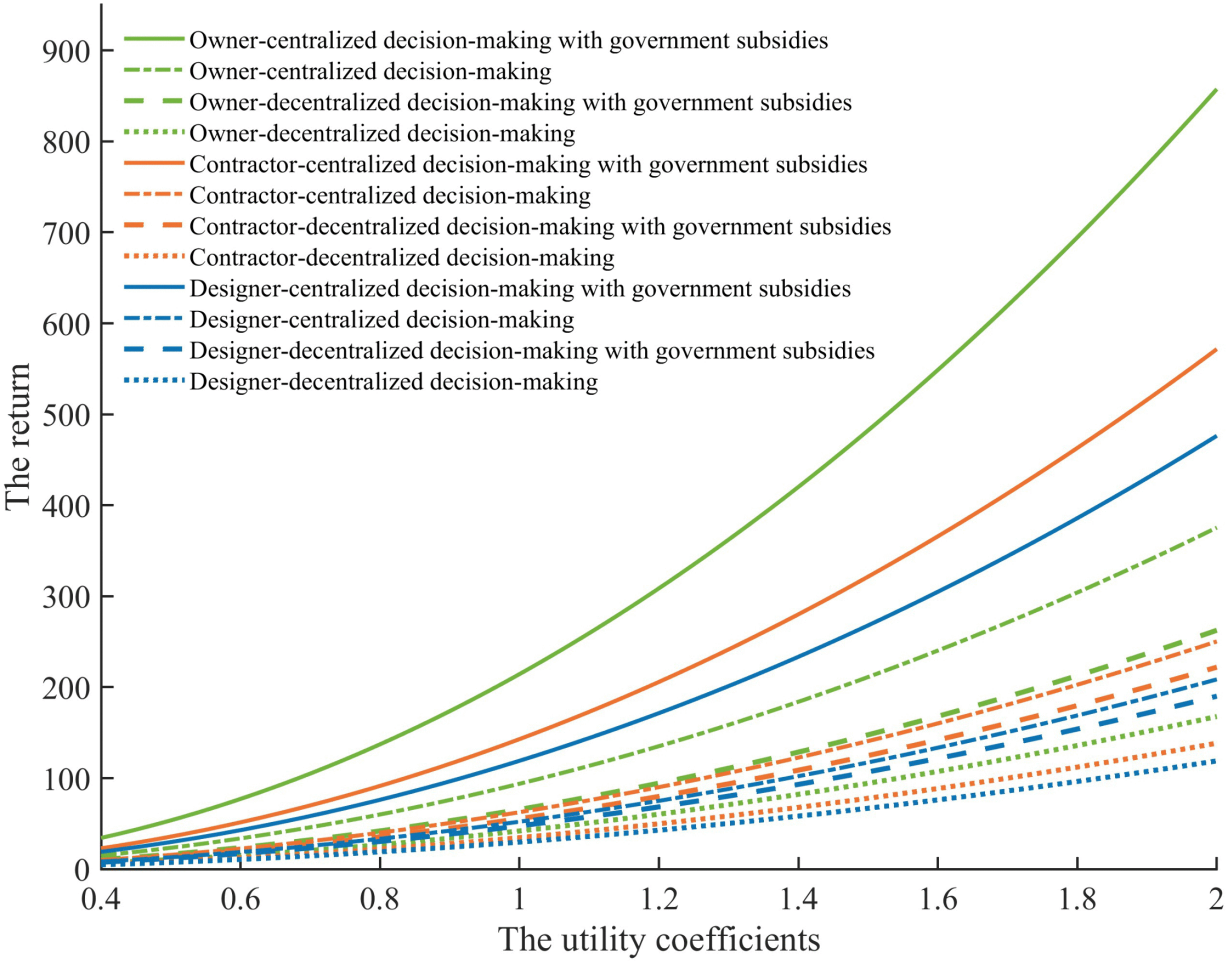
Impact of utility coefficients of contractor, designer and owner on their returns.

The effects of the utility coefficients of contractor, designer, and owner on their effort levels and returns are shown in Figs 10–11.

Figs 10–11 show that increases in the utility coefficients significantly enhance the effort levels and returns of all parties. This indicates that the stronger the ability to convert data value into returns, the more willing the parties are to participate in data value co-creation.

### 5.7. Robustness analysis

**Fig 12:**
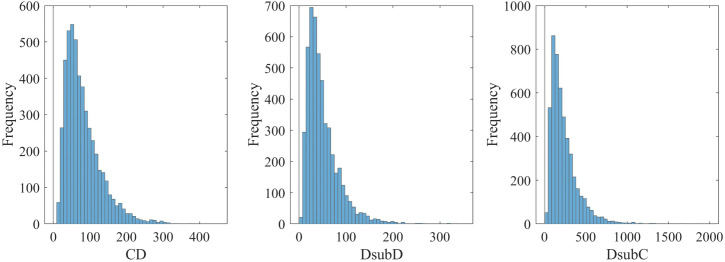
Robustness analysis of return differences under different decision scenarios.

To examine the stability of the model results, this study further conducts robustness analysis. Based on the baseline parameters in Table 2, the parameters are varied within a certain range and randomly generated into parameter combinations, and the changes in return under different decision-making scenarios are compared, as shown in Fig 12.

In Fig 12, CD denotes the difference in returns between the centralized and decentralized decision-making scenarios. DsubD $\Delta_{\text{2}}$and DsubC $\Delta_{\text{3}}$represent the changes in returns caused by subsidies under the decentralized and centralized scenarios, respectively. Fig 12 shows that the return differences of all samples lie to the right of zero, indicating that the differences are consistently positive. The results suggest that, under different parameter combinations, the total return under centralized decision-making is always higher than that under decentralized decision-making. And the government subsidies further enhance the effort levels of the parties and the overall return. Therefore, the model conclusions do not depend on specific parameter values and exhibit strong robustness.

## 6. Discussion and policy implications

### 6.1. Discussion

This study constructs a dynamic game model of data value co-creation under four decision-making scenarios involving contractor, designer, and owner during the construction of water conservancy projects. The study finds that decision-making mode, government subsidies, cost coefficients of data value co-creation, and utility coefficients have significant impacts on data value co-creation results. From the perspective of decision-making modes, water conservancy projects are characterized by long construction cycles, multiple participants, and dispersed data. Centralized decision-making reduces information asymmetry and strategic conflicts among parties through a unified coordination mechanism, promotes data resource integration, and thereby improves the efficiency of data value co-creation. Government subsidies further enhance the willingness of the parties to participate in data value co-creation by reducing their investment costs and strengthening collaborative incentives. Although previous studies have confirmed the role of government policy incentives [50], such incentives play a particularly important role in promoting data value co-creation in water conservancy projects, where government leadership is relatively strong. In addition, the cost of data value co-creation and the utility coefficients influence the decision-making behavior of the parties. When collaboration costs are high, the parties tend to reduce their inputs, which is consistent with the findings of An et al. [38]. By contrast, when the efficiency of converting data value into actual benefits improves, the willingness of the parties to participate in data value co-creation increases significantly.

Compared with previous studies [51,52], this study constructs a dynamic decision-making model for multi-party data value co-creation by incorporating the characteristics of water conservancy projects. The equilibrium strategies of data value co-creation under different decision-making scenarios and their influencing factors are analyzed. The results extend research on data value co-creation in the context of water conservancy projects. They also provide theoretical support for improving the efficiency of data element utilization and enhancing the quality and efficiency of water conservancy project construction.

### 6.2. Policy implications

Based on the research findings, several policy implications are proposed to further promote data value co-creation in the construction sector.

(1) Establish a centralized decision-making mechanism for data collaboration. The results indicate that centralized decision-making can significantly enhance multi-party data value co-creation. Therefore, during the construction of water conservancy projects, the owner or the government can take the lead in establishing a unified data management platform to promote data sharing among participants, such as designers and contractors, thereby improving multi-party collaborative decision-making capabilities.
(2) Establish a differentiated government subsidy mechanism. The results indicate that government subsidies significantly enhance multi-party data value co-creation, with stronger incentive effects under centralized decision-making. Therefore, the government may set differentiated subsidy rates based on the level of data resource investment by different participants and actively promote centralized decision-making among them, thereby strengthening collaborative participation in data value co-creation.
(3) Reducing the cost of data value co-creation to enhance the effort levels of participating parties in data value co-creation. An increase in the cost of data value co-creation significantly inhibits the willingness of each party to participate in data value co-creation. Therefore, efforts should be made to reduce overall costs by improving data standard systems, optimizing data management processes, and promoting digital technologies such as BIM and digital twins. This can reduce redundant data collection and the costs of data sharing and utilization, thereby lowering the overall cost of data value co-creation and enhancing the willingness of participating entities to engage in data value co-creation.
(4) Improving data value conversion efficiency to enhance the motivation of participating parties. The application of project data in design optimization, construction planning optimization, and quality and safety management should be strengthened. Artificial intelligence technologies can be leveraged to further explore the potential value of data and promote the transformation of project data into practical benefits, thereby enhancing the willingness of all entities to participate in data value co-creation.

## 7. Conclusion

The construction of water conservancy projects generates vast amounts of data, the effective utilization of which can enhance project benefits. Data value co-creation is a critical pathway to realizing data value. Based on differential game theory, this study constructs four data value co-creation game models under different scenarios, and analyzes the optimal strategies for participating subjects in data value co-creation and the impact of key factors on these strategies. The main conclusions of this study are as follows:

(1) Centralized decision-making is more conducive to promoting multi-party data value co-creation in water conservancy projects. During water conservancy project construction, data are dispersed among multiple parties, such as designer, contractor, and owner. Centralized decision-making facilitates data sharing and resource integration through a unified coordination mechanism, thereby significantly improving the benefits of data value co-creation.
(2) Government subsidies can effectively incentivize multiple parties to participate in data value co-creation, with stronger effects under centralized decision-making. Water conservancy projects are characterized by strong government leadership. Appropriate subsidy mechanisms can reduce the data collaboration costs of participating parties and promote multi-party cooperation, thereby further enhancing the benefits of data value co-creation.
(3) The cost coefficient of data value co-creation has a significant inhibitory effect on the data value co-creation behavior of participating parties. As the cost coefficient increases, the effort levels and returns of all parties decrease. During the construction of water conservancy projects, reducing the costs of data collection, integration, sharing, and utilization can enhance the willingness of parties to participate in data value co-creation.
(4) An increase in the utility coefficients can significantly improve the effort levels and returns of participating parties in data value co-creation. When data value can be more effectively converted into benefits, the willingness of each party to participate in data value co-creation is significantly enhanced. Improving the efficiency of data value conversion is an effective way to promote participation in data value co-creation.

This study provides a new perspective for exploring the value of data elements in water conservancy projects. The findings offer theoretical support for data value co-creation practices and related policy formulation in water conservancy projects. Through data value co-creation, the value of massive data elements in water conservancy projects can be activated, thereby improving project performance. However, the foundation of data value co-creation lies in the reasonable sharing of co-created value benefits. This study merely presents this viewpoint in the research process and does not conduct in-depth research on the benefit-sharing mechanism of data value co-creation, which will be a direction for future research.
